# Multidisciplinary perspectives to prevent occupational health-related conditions among dental practitioners

**DOI:** 10.1038/s41405-019-0010-3

**Published:** 2019-04-11

**Authors:** Rajeshree Moodley, J. Van Wyk

**Affiliations:** 1 Discipline of Dentistry, School of Health Sciences, University of KwaZulu-Natal, KwaZulu-Natal, South Africa; 2 Discipline of Clinical and Professional Practice, School of Clinical Medicine, College of Health Sciences, University of KwaZulu-Natal, KwaZulu-Natal, South Africa

## Abstract

**Introduction:**

The prevalence of occupational health conditions is high among dental practitioners and this study investigated the role which occupational health plays in dental training.

**Purpose/objectives:**

This study was conducted to explore occupational health and to determine the topics to include from an occupational health perspective into the dental curriculum.

**Methods:**

A descriptive qualitative study was conducted to explore the perceptions of dental practitioners, dental academics, physiotherapists, occupational therapists, occupational health specialists, ergonomists, optometrists and audiologists about dental training from an occupational health perspective in KwaZulu- Natal, South Africa. The interdisciplinary and multidisciplinary approach was used in this study.

**Results:**

Three main themes became evident that hinged on varying understanding of occupational health-related conditions to dental practice, how practitioners experience practising in the resource-poor settings and its impact on the dental practice. There was also a lack of awareness of the occupational health policies and practices, which could inform safe dental practice.

**Conclusion:**

Dental academics should gain input from a multidisciplinary team. An occupational health course with a student-centred approach would enrich the dental curriculum and make dental practitioners more aware of occupational health issues.

## Introduction

The number of dental practitioners in South Africa (SA) is 12,904, which consists of 9541 dentists, 1094 dental therapists and 2269 oral hygienists serving a population of 55.91 million people.^1^ Eighty-six percent of the dentists in SA are employed in the non-public or private sector.^2^ There is an unequal distribution of dental practitioners within the public and private sector placing practitioners in the public sector at risk of occupational health-related conditions due to a higher workload and patient volumes. There is a need for dental practitioners to be free of occupational health-related conditions so that they can be productive, healthy and serve the population for a longer time.

Dental practitioners are at risk of various occupational health-related conditions. The systemic diseases of concern in the South African setting specifically relates to HIV, TB, hepatitis C and hepatitis B. In addition, they are exposed to allergies including monomers, latex and solvents that cause dermatitis and respiratory diseases. They are also exposed to physical hazards due to radiation and noise. A previous study on dental practitioners in KwaZulu-Natal, South Africa reported on ergonomic factors that impacted on practitioners’ health that included musculoskeletal disorders, carpal tunnel syndrome and other overuse disorders.^3^ That study highlighted how a lack of awareness of occupational health principles and safety impacted on the health and productivity of dental practitioners. Occupational health is defined by the World Health Organisation as “all aspects of health and safety in the workplace and has a strong focus on primary prevention of hazards”.^4^

Pain is also prevalent among dental students. Even more disconcerting was the finding that revealed the prevalence of pain among dental students and the fact that the percentage of pain among students steadily increased from the first (41%) to their fourth year (71%).^5^ Incorrect postures with sudden flexure of the neck and cervical twisting puts students at risk of muscle pain.^6^ Eighty percent of dental students at the University of Cartagena, South America reported pain due to clinical practice.^6^ Female students were found to be at a higher risk of developing pain.^5,6^ Researchers in a South African study reported a high level of stress among dental students with stress levels peaking in the fourth year of study.^7^

Statistics among students and young practitioners indicate a need for greater awareness in dental curricula to prevent occupational health-related conditions among dentists. It is thus clear that training about primary prevention of the many occupational hazards is a necessity to ensure that students become more aware of their body position and work habits.^5^

Occupational health-related conditions are prevalent among dental practitioners. Stress, musculoskeletal disorders and percutaneous injuries are issues that are common among both young and experienced dental practitioners. To include occupational health in a dental curriculum required perspectives of various disciplines and in this study, the researcher engaged in a focus group discussion to get multi-stakeholder input into the dental curriculum. Physiotherapists, occupational therapists, occupational health specialists, ergonomists, optometrists and audiologists were also part of these discussions. This team would input from a coordinated range of skill, expertise and clinical experience as discussed by Young, 1998 who further stated that such a team allows input for a common goal.^8^

Focus group discussions were conducted to determine what could be included in the dental curriculum to make dental students aware of self-care in terms of their occupational health. The interdisciplinary and multidisciplinary approach was used in this study. A multidisciplinary approach to dental education would be to draw from different academic disciplines to redefine an approach or understanding and, in the case of this study, it would be to design a course by getting multidisciplinary input.^9^ The rationale for the use of this approach is that occupational health-related conditions are treated by a team of health care workers and their input was required.

The dental curriculum prepares a student for work placement and traditionally dental curricula focuses on the core competency of graduates. Education and training is vital to ensure safe and healthy working. Alli, 2001 suggested that health and safety principles be incorporated into student training relating to the needs of the profession.^10^

One of the major health issues that emanates from students as they progress in academic  years is stress, which impacts on students’ and dental practitioners’ well-being. A study conducted in a dental school in Karnataka, India reported that stress levels among dental students showed a marked increase from first to the fourth year with stress peaking in the second and third year. Fear of failure was the highest stressor.^11^ Similar results can be seen in the Jordanian study where fourth and fifth-year students displayed higher levels of burnout.^12^ Senior students in a Saudi Arabian study showed higher levels of stress than first-year students.^13^ For students at a South African University, stress peaked in the fourth year (47.9%) with stressors including lack of effective teaching/lectures in the first year (49%) and lack of motivation in the second year (39.2%). A lack of student input into management decisions and the non-response by management to the needs of students were issues with fourth and fifth year students (40 and 50%).^7^ The research displays that there is a need to teach stress prevention at universities. It needs to be reinforced at universities where it is already being done.

This study, reports on a qualitative case study conducted in the University of KwaZulu-Natal, South Africa to elicit the occupation-related challenges experienced by dental practitioners in dental practice. This study also provided recommendations for a multidisciplinary team including other health care workers. The input included specific curriculum content/topics to ensure a more occupationally responsive dental training programme.

## Research methods

Ethical clearance was obtained from the Humanities & Social Sciences Research Ethics Committee University of KwaZulu-Natal (UKZN)—reference no: HSS/1490/015D.

A descriptive qualitative study was conducted to explore the perceptions and challenges of dental practitioners, dental academics, physiotherapists, occupational therapists, occupational health specialists, ergonomists, optometrists and audiologists about dental training from an occupational health perspective.

All the practitioners were selected for having had more than five years of clinical experience in dental practice or in practice in their own field. A purposive sampling strategy was used to recruit participants for the focus group discussions. Four focus group discussions were conducted between May and June 2017. A total of 36 participants participated in the four focus group discussions. The participants in focus group one consisted of a group of dental practitioners all who had experienced an occupational health problem (*n* = 7). The participants of focus group two consisted of a team of multidisciplinary professionals (*n* = 9) representing physiotherapists, occupational therapists, occupational health specialists, ergonomists, optometrists and audiologists. The third focus group consisted of dental practitioners with clinical experience and clinical supervision experience (*n* = 13). The last group consisted of dental academics who had been involved in clinical supervision, curriculum development and who taught on the undergraduate programme (*n* = 7). A facilitator conducted the focus groups while the researcher played the moderator’s role to reduce bias. The focus group used semi-structured open-ended questions. The discussions centred on personal experiences, experiences in academia, challenges in dental training and recommendations for dental training and curriculum content. Each focus group discussion lasted between 60 and 90 min. This qualitative approach using semi-structured focus group discussions rather than questions allowed us to investigate broader areas of concern and was relaxed as it was semi-structured. In the end, the participants themselves gave positive input about the focus group. In addition three interviews were conducted with stakeholder participants who were unable to attend the discussions of their particular focus group and the data were added to that collected from the group.

This phase of the study was part of a larger study that investigated the role of occupational health in dental training. Ethical clearance was obtained from the Humanities & Social Sciences Research Ethics Committee (UKZN)—reference no: HSS/1490/015D. All participants were informed of the study through an information letter; they completed an anonymous demographic sheet and signed written consent prior to participation. They also consented to an audio recording. They were given the option to withdraw from the study at any stage. The anonymity of participants was assured and maintained throughout the study by using participant codes instead of names. All data were kept in a locked password protected e-file.

The audio recordings were transcribed verbatim and checked for accuracy. The transcripts were sent to a participant in each focus group for member checking or respondent validation. This was conducted to establish rigour and validity.^14^ The data were then thematically analysed. All transcripts were read and the researchers made written notes and noted patterns. Broad themes were identified, according to the main aim, then further refined and coded after which the final analysis was done.^15^

## Results and discussion

The demographic details of the participants are summarised in Table 1. All participants (*n* = 36) were qualified with an undergraduate degree (in either health science, dentistry, dental therapy or oral hygiene) and some participants had postgraduate qualifications (*n* = 14). The table shows the years of experience as a practitioner. The average age of the participants was 40–50 years. Most of the participants had previous experience in curriculum development with some reporting experience also in curriculum review. Three main themes emerged in response to the questions explored (as indicated in Table 2). The themes were; varying understanding of occupational health-related conditions to dental practice and how practitioners experience practice in the resource-poor settings and its impact on the dental practice. There was also a lack of awareness of the occupational health policies and practices, which could inform safe dental practice.

**Table 1:** Demographic information and experience (teaching and clinical) of participants

	Average age	Profession	Post held	Qualification	Average years of experience as a practitioner	Average years of experience as an academic	Involvement in curriculum design
Focus group 1- dental practitioners with occupational health problems (*n* = 7)	40.7	Dentist, dental therapist and oral hygienists	Lecturers and clinicians	Undergraduate degree (*n* = 5) Master’s degree (*n* = 1) PhD (*n* = 1)	19	6.7	Two participants had experience with curriculum design and development
Focus group 2- multidisciplinary team (*n* = 9)	43.7	Optometrist, physiotherapist, ergonomist, audiologist, occupational therapist, dentist, dental therapist, dentists	Lecturers, senior lecturers, professors and clinicians	Undergraduate degree (*n* = 2) Master’s degree (*n* = 2) PhD (*n* = 5)	20.8 years	12.1	All participants except for one had experience with curriculum design and development. Three participants were involved with curriculum reviews at other universities
Focus group 3- dental practitioners (*n* = 13)	41.6	Dentists, dental therapist and oral hygienists	Dentists, dental therapists and oral hygienists that have honorary clinical supervision posts	Undergraduate degree 9 (*n* = 13)	17.5	All participants are clinical supervisors with 2 having teaching experience	Two participants had experience with curriculum design and development
Focus group- 4 dental academics (*n* = 7)	49.1	Dentists, dental therapist and oral hygienists	Senior tutors, lecturers and senior lecturers and clinical supervisors	Undergraduate degree (*n* = 2) Master’s degree (*n* = 2) PhD (*n* = 3)	23.1	12.1	All participants had experience with curriculum design and development. Two participants were involved with curriculum reviews at other universities

**Table 2:** Themes emanating from focus group discussion

Main theme	Sub-themes	Description	Quotation/supporting evidence
Challenges experienced in dental practice and training	Understanding occupational health. This	There were different views on what occupational health entails	“pain relating to the work that you basically do” (focus group 4, participant 1) “the first thing that comes to your mind when you hear occupational health, that it is not just not purely physical it is the psychosocial aspects and that congruence between what you experience physically and what you experience psychologically.” (focus group 3, participant 2)
		There was a lack of holistic understanding of what occupational health entails	“So, I understand it to be the procedures we do, the physical activity and how it affects your quality of life. That’s what I perceive it to be” (focus group 1, participant 1) “I never really thought about it until I started working, and then I kind of wised-up in terms of my posture, my surroundings” (focus group 1, participant 2)
		Lack of understanding occupational health risks and hazards inherent in the dental practice	“I would say it is divided into two it is strictly work-related and strictly human factor related. For the work-related it seems your chair your equipment…” (focus group 4, participant 5)
		Perception that muscoloskeletal disorders (MSD) was the only occupational hazard	“I think for me I just consider the physical.” (focus group 3, participant 3)
	Dental practice in SA. Some were aware that high-income countries instituted better policies.	First world versus third world. Resource-poor settings were experienced as very limiting. Lack of equipment and resources in low-income countries	“When I moved to Australia I worked in a clinic there where part of our regime was at quarter to ten you would stop working and you would take your tea break and do stretching exercises. Just stretching and then before lunch we do the same before you went on lunch and that was enforced even if you had a patient you had to stop go and do you exercise.” (focus group 4, participant 5) “Sometimes we use the visors for week, two weeks. I had a visor for like three weeks and I couldn’t even see properly.” (focus group 4, participant 5)
	Dental Training under resource restricted conditions	Lack of resources is common as dental training or dentistry is not seen as priority in SA	“We start off wrong and that is the biggest problem. This is where it starts when you are not sitting properly everything else goes wrong because it is your positioning because we have chairs that are not working properly not adjustable properly they do not roll properly…” (focus group 4, participant 5) “I also agree with what……. is saying is that if you want to make something a habit the environment has to be conducive. So, where we are teaching it not in the pre-clinic there we have typists, chairs and the one head will be right down and the other head on the top. So, we are teaching them in a wrong environment if we have the right equipment.” (focus group 4, participant 1)
	Lack of awareness of occupational health policy/principles	Participants do not know if principles and policies were in place.	“what lacks in private practice is policy and procedure” (focus group 1, participant 6) “Because only after I went through everything did I realise there’s a huge manual on what constitutes a hazard and repetitive strain injury. And I ticked every box and I thought oh my god, I have been through all this and I didn’t know this manual existed.”

Practitioners also offered recommendations for curriculum content about self-care and occupational health to include during dental training (as indicated in Table 3). Table 3 also includes the sub-themes, a basic description and the supporting evidence.

**Table 3:** Recommendations for dental training

Main theme	Sub-themes	Description	Quotation/supporting evidence
Recommendations for dental training and curriculum content	Recommendations for curriculum	Participants added what they thought should be included in dental training	“Yes, so I think for me, the first step would be to get an idea of exactly how these working environments are set up. So, to have an understanding of how these working activities work, so we call that at-risk assessment. So that’s for a person who is going into a ready-made practice. But for someone who’s starting out, I’d certainly like them to have training in how to set up the optimal work environment. So, you make sure upfront that there will be no issues. For example, if you’re going to be mixing amalgam, you make sure it is done in a separate area that is adequately ventilated. If you are using noxious gases, you use it under controlled conditions. Either you have protection, if the levels are low or some extractive ventilation. So, the first step is understanding, or redesigning the work environment or designing it in a way that these are taken into consideration. It’s far better to build from scratch, bearing this in mind, than trying to fit the work to the premises. It requires more money etc. so that’s the first step. I would like dental professionals to get a sense of the risks in what they do and what health outcomes are associated with these risks are. If they can understand those three concepts—risk assessment, specific risks and broad health outcomes—they would have a better understanding of how these can be addressed” (interview one-occupational health specialist)
	Recommendations for clinical practice	Should occupational health be a stand-alone module or incorporated into existing modules?	“I think it should be a module on its own—occupational hazards. Like 10 or 20 sessions and in each session, talk about a hazard” (focus group 1, participant 3) “Firstly, I do not think there is a need for a module that is stand-alone because it is going to be based on everything else where the student will want to pass that module” (focus group 2, participant 5) “So sitting position is important and I would add to that some back exercises lower back exercises, the back to me is very important.” (focus group 4, participant 5)

The combined input of dental practitioners from multiple disciplines was beneficial in the consideration of a dental programme including occupational health principles. From the focus group discussions, it became clear that participants strongly supported the inclusion of an occupational health course. They suggested that it be introduced in dental training and for an interdisciplinary approach to be followed during the curriculum design process. Participants of focus group one recommended that input is sought from audiologists, occupational therapists, physiotherapists, ergonomists and occupational health specialists.

## Challenges

### Understanding occupational health

One of the participants highlighted the psychosocial aspect of occupational health. Both physical and psychosocial stressors are prevalent in dentistry.^16^ The participants highlighted the physical aspects of dental practice. Participants of this study, had a perception that musculoskeletal disorders (MSD) was the only occupational related health condition among dental professionals. An explanation for this is that MSD is the most prevalent and most reported among dental practitioners. This can be seen in Phase 2 of this larger study where the occupational health-related problems were investigated among dental practitioners in KwaZulu-Natal South Africa, where MSD has the highest prevalence among the occupational health-related issues.^17^ To reduce the prevalence there must be a change in dental training as MSD symptoms start in dental school and progress into practice.

### Dental practice in South Africa

The focus group three discussions revealed that dental training prepares students for private practice and not public practice. This was seen in an American study where the researchers recommended that more topics related to public health and practice be included in the dental training.^18^ There were mixed feelings on whether dental training should prepare students for public practice as most of the students are employed in the private sector. Those who are employed in the public sector faced with high patient volumes for which they were not prepared causing stress. According to Baldwin et al.,^19^ there is a significant difference between those working in public and private practice in terms of workloads and financial burden. Stress management should be introduced at first-year level so that students learn to manage their stress.

### Dental training in South Africa

#### Recommendations

An occupational health medical at the beginning of preclinical years will make students aware of the prevention of occupational health problems and should be followed twice a year when employed.^20^ Dental curriculum includes posture in preclinical years as seen in the results of these focus group discussions. Clinical supervisors pay attention to the tasks at hand rather than the posture of the student. The recommendations from dental practitioners in this study were to include a course on occupational health in the final year of training while there should be a link in each clinical module with clinical assessments. The patient position in relation to the operating position is important in the prevention of carpal tunnel syndrome. The hand and elbow in relation to shoulders should be lower. Stretching of the body and rotation of hips while working predisposes the operator to MSD. The inclusion of the causes of MSD, the muscles involved, the physiology of this kind of pain and its prevention should be included in this possible module. The selection of dental instruments, weight and grip influence the type of pain experienced by dental practitioners and should also be included.^21^ A practitioner will select instruments based on funding rather than what is ideal. Feng et al.^22^ concluded in their study on the prevalence of work-related musculoskeletal symptoms of the neck and upper extremity among dentists that the inability to select new instruments places dental staff at a higher risk of shoulder, wrist and hand pain Hand injuries are due to high pinch forces when working with a gloved hand and the textured appearance of dental instruments plays a role in the force applied to the instrument which invariably affects the grip.^23^ Regular aerobic, stretching and strengthening exercises should be undertaken to strengthen the musculoskeletal system. Dental students should be taught self-care as the research has shown that regular training can prevent MSD.^24^

The occupational health specialist in this study recommended that the work environment must be optimal and all risks must be avoided. He suggested that if students understood the concepts of risk assessment and workplace design, then they would be able to prevent work-related conditions. The study conducted by Khan and Chew^25^ recommended that theory and practice of ergonomics should be taught in dental schools as their study, which was conducted in Malaysian dental schools showed a 93% prevalence of MSD among the student participants. Good ergonomic practices can prevent MSD and should be introduced in the early years of training.^3,26^ Ergonomic training should focus on workstation design, physical job features, lifting, awkward postures and repetitive tasks.^27^

Dental practitioners should mobilise their job resources and stay engaged in their work. Practitioners with good job control and those who constantly improve their work by being innovative are the ones who best deal with their job demands. The researchers in the Hakanen study revealed that how to stay engaged in your work is something that should be taught in undergraduate training. They also identified that open communication with clients and colleagues, variability of required skills and how to increase resources in practice should be included in dental curricula.^28^

#### Clinical practice

Safe work practice and infection control were highlighted and discussed in this focus group discussion. It was recommended by Alsabaani et al.,^29^ that safe work practice be emphasised in the early years of dental education. The use of magnification should be introduced in the first year of training and reinforced in all years of undergraduate training. First-year students should be familiar with eye care, have regular eye checks and understand the prevention of eye injuries from level one. Eye safety and eye care awareness are compulsory in dentistry as dental practitioners work with sharp and rotary instruments. The splashes created have blood, saliva, particles of teeth, restorative materials and oils from the headpiece and micro-organisms. This leads to injuries when the eye is unprotected.^29–31^ When wearing eye protection, these injuries do not occur but unfortunately compliance with eye protection is poor.^32,33^ Dosimeters to monitor radiation exposure levels should be introduced.^34^ Staff and students in dental radiography currently do not use dosimeters. When one draws from Singh’s article, a single accidental exposure to high radiation may produce biological effects after exposure.

## Limitations of the study

The study population is not representative of the dental practitioners and academics of South Africa but more information was gathered than in a cross-sectional study. Another limitation of a focus group discussion is that the views of one participant may affect the views of others.^35^

## Conclusion

The results of the present study highlight the need to review dental curriculum and to redesign curriculum to feature occupational health. Dental academics should gain input from a multidisciplinary team. An occupational health course with a student-centred approach would enrich the dental curriculum and make dental practitioners more aware of occupational health issues. Occupational health and self-care should be included in health science training in general and not only in dentistry. Further research is required in this area. Further research should be conducted to gain the input of students into a curriculum that incorporates occupational health.
